# Floral Fusion: Unravelling the Potent Blend of Ixora coccinea and Rhododendron arboreum for Health and Safety Benefits

**DOI:** 10.7759/cureus.70038

**Published:** 2024-09-23

**Authors:** Pavithra Bharathy, Punniyakoti V Thanikachalam, Arundhamizh N Shoban, Harish V Himayavendhan

**Affiliations:** 1 Department of Pharmaceutics, Saveetha College of Pharmacy, Saveetha Institute of Medical and Technical Sciences, Saveetha University, Chennai, IND; 2 Department of Pharmaceutical Chemistry, Saveetha College of Pharmacy, Saveetha Institute of Medical and Technical Sciences, Saveetha University, Chennai, IND

**Keywords:** embryonic toxicity, inflammation, ixora, oxidant, rhododendron, zebrafish

## Abstract

Objective: *Ixora coccinea* and *Rhododendron arboreum* are known for their traditional medicinal uses due to their diverse phytochemicals and pharmacological effects, which have attracted the interest of many researchers. This study aims to evaluate the antioxidant, anti-inflammatory, and cytotoxic effects of their combined extracts.

Methods: In vitro antioxidant activity against reactive oxygen species (ROS) was measured using the ferric-reducing ability of plasma (FRAP), nitric oxide (NO), and 2,2-azino-bis-3-ethylbenzothiazoline-6-sulphonic acid (ABTS) assays, while anti-inflammatory effects were assessed via the membrane stabilization method. Docking studies were performed to evaluate the interaction of phytochemicals - anthocyanins, quercetin, and ursolic acid, which are present in these plants - with nuclear factor kappa B (NF-κB), cyclooxygenase-1 (COX-1), and cyclooxygenase-2 (COX-2). Standard protocols were used to evaluate embryotoxicity using the brine shrimp model and cytotoxicity using the zebrafish model, which is crucial for determining safe clinical dosages.

Results: The analysis revealed diverse bioactive compounds, including anthocyanins, quercetin, and ursolic acid. The formulation effectively inhibited ROS production at lower concentrations (inhibitory concentration 50%, or IC50 value ~2.8 µg/mL), indicating their potential for managing oxidative stress. Quercetin demonstrated the strongest interaction with all tested proteins, particularly NF-kB. Cytotoxicity and embryotoxicity assays revealed a dose-dependent effect (lethal concentration 50%, or LC50 value 82.4 µg/mL), with no adverse effects on developing embryos at the tested doses (5-80 µg/mL), suggesting the extracts are safe for clinical use, even during pregnancy.

Conclusion: The combined extracts of *I. coccinea* and *R. arboreum* exhibit potent antioxidant and anti-inflammatory effects without causing cytotoxic or embryotoxic effects, even at higher concentrations. This indicates their potential for safe clinical application in treating oxidative and inflammatory diseases.

## Introduction

*Ixora coccinea* L., commonly known as Burning Love or Jungle Geranium, is a short evergreen shrub prevalent throughout Asia. Other *Ixora* species, such as *I. chinensis, I. fulgens, *and *I. polyantha*, also have medicinal value. *I. chinensis*, with nearly stalkless leaves and pink flowers, has been used for stomach issues, tuberculosis, and headaches [1]. *I. coccinea*, identified by its red flowers, grows in abandoned fields and is rich in phytochemicals such as anthocyanins, fatty acids, saponins, and tannins [2]. Traditional uses include treating diarrhoea, fever, ulcers, bronchitis, and dysentery. Its wound-healing, cytotoxic, antimicrobial, anti-inflammatory, and antioxidant activities are well-documented [3,4].

*Rhododendron*, a genus native to the Northern Hemisphere, has been noted since 401 B.C. for its toxic honey. Despite its toxicity, many cultures, including mediaeval Chinese and Ayurvedic traditions, have used it in folk medicine [5]. Research shows *R. arboreum* flower extracts have cardioprotective properties, with fractionated extracts showing high potentials [6]. Ethnopharmacological studies emphasize its therapeutic potential and toxicity, providing a foundation for future drug development [7].

Reactive oxygen species (ROS), products of normal cellular metabolism, can damage lipids, proteins, and DNA. In the Fenton reaction, hydrogen peroxide reacts with transition metal ions, producing hydroxyl radicals (•OH), among the most reactive ROS. ROS are vital for host defence and cell signalling, but imbalances can cause oxidative stress linked to diseases like cancer, heart disease, and neurological disorders [8].

Embryonic toxicity assays assess potential harm to developing embryos or fetuses, detecting teratogenic effects or developmental defects. Regulatory agencies, including the European Medicines Agency and the US Food and Drug Administration, require such tests for drug approval. Studies show the ethanol extract of *Curcuma zedoaria* has lethal concentration 50% (LC50) values of 588 parts per million (ppm) and 224 ppm against brine shrimp larvae and zebrafish embryos, respectively [9-11]. Extracts of *Ambrosia tenuifolia* demonstrated dose-dependent toxic effects in zebrafish embryos, while polydatin showed no toxicity up to 435 μM [12,13].

In this study, we assessed the antioxidant potential of a formulation containing *I. coccinea* and *R. arboreum*, focusing on its ability to inhibit ROS and stabilize cell membranes under stress. Additionally, embryonic toxicity and cytotoxicity assays were performed to determine safe dosing levels, which are essential for evaluating the formulation’s safety and guiding its application in future clinical trials.

## Materials and methods

Collection and preparation of extract

**Figure 1:**
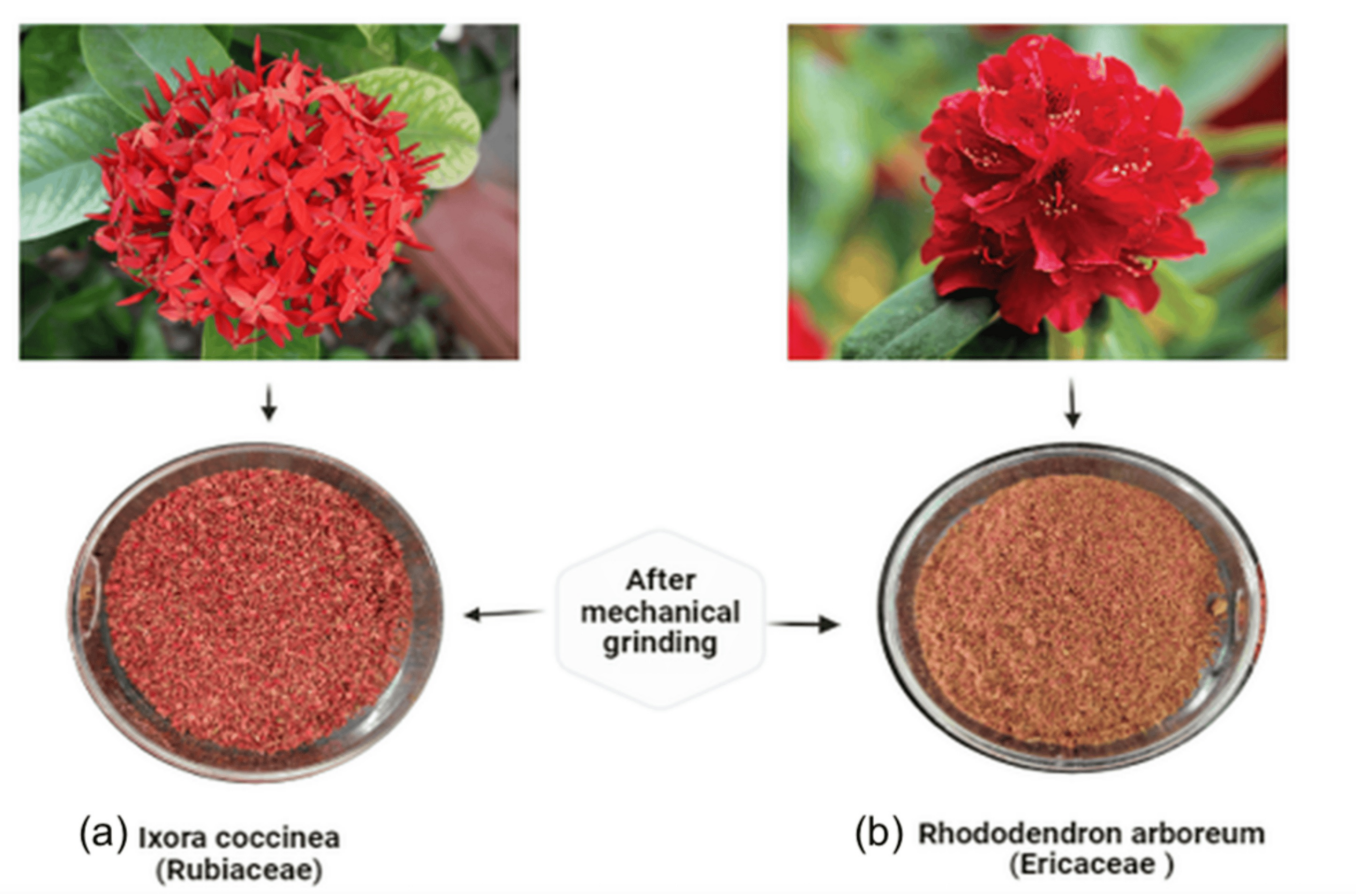
Conversion process of red flowers to powdered form, including Ixora coccinea (a) and Rhododendron arboreum (b). The process starts with the flowers in their natural state, followed by drying to remove moisture. The dried flowers are then ground into a coarse powder, which is subsequently sieved to obtain a fine, uniform powder.

*R. arboreum* and *I. coccinea* flowers were sourced from natural herb traders in Almora, Uttarakhand, and P.K.M. flower suppliers in Tiruchirappalli, Tamil Nadu, India, respectively. Disease-free flower parts were shade-dried for seven to eight days, ground into fine powder (Figure 1), and subjected to solvent extraction. For ethanolic extraction, 100 g of dried flower tissues were packed in a Soxhlet apparatus with 1000 mL of ethanol for eight hours. The resulting extract was concentrated and dried, yielding approximately 4.02% weight/weight (w/w) of Rhododendron ethanolic extract (RhE) and 5.3% w/w of Ixora ethanolic extract (IxE). For aqueous extraction, 100 g of shade-dried powdered flowers were submerged in 1000 mL of water with 5 mL of chloroform as a preservative. The maceration process lasted for 72 hours with periodic shaking, yielding a brownish-red watery extract. After filtration and concentration, the aqueous extract yields were approximately 3.18% w/w for Rhododendron water extract (RhW) and 4.06% w/w for Ixora water extract (IxW). A combination of equal proportions (1:1) of 100 g of *Ixora* and *Rhododendron* flowers underwent ethanolic extraction (IRE) using the same method, resulting in a yield of approximately 9.18% w/w.

Quantitative phytochemical analysis

Quantitative analysis was conducted for the phytochemical components, the presence of which had been previously tested and confirmed by qualitative analysis for *R. arboreum* extracts (RhE and RhW) and *I. coccinea* extracts (IxE and IxW), as described earlier [14,15].

Alkaloid determination

For quantitative estimation of alkaloid content, 2 mL of HCl was added to the extract dissolved in ethanol and 5 mL of phosphate buffer (pH 4.7). Then, 5 mL of bromocresol green and 4 mL of chloroform were added and mixed thoroughly. The absorbance of the diluted extract solution containing chloroform was measured at 470 nm with atropine (AT) as a standard. The AT equivalent was used to calculate the alkaloid content (mg AT/g).

Phenolic determination

After diluting 2 mL of the extract with distilled water, 0.5 mL of the Folin-Ciocalteu phenol reagent was added to estimate the quantitative phenolic content. Add 2 mL of 35% sodium carbonate and incubate at room temperature for 20 minutes. The absorbance of the diluted extract solution with distilled water was measured at 750 nm with Gallic acid (GA) as a standard. The GA equivalent was used to calculate the phenolic content (mg GA/g).

Flavonoid determination

For quantitative estimation of flavonoid content, 1 mL of 5% sodium nitrite and 3 mL of 10% aluminium chloride were added to 2 mL of extract and stabilized for five minutes. Add 2 mL of sodium hydroxide to the above mixture, and make up the volume to 10 mL and incubate at room temperature for six minutes. The absorbance of the diluted extract solution with distilled water was measured at 510 nm with catechin (CN) as a standard. The flavonoid content was determined as CN equivalent (mg CN/g).

Saponin determination

For quantitative saponin content estimation, 2 mL of vanillin reagent and 3 mL of 72% sulphuric acid were added to 2 mL of extract, mixed well and heated in a water bath at 60°C for 10 minutes. The absorbance of the diluted extract solution was measured at 544 nm with diosgenin (DI) as a standard. The saponin content was determined to be DI equivalent (mg DI/g).

Tannin determination

For quantitative estimation of tannin content, 1.5 mL of extract was diluted with 6.5 mL of distilled water, followed by 2 mL potassium ferricyanide and 2 mL ferric chloride were added. The above mixture was diluted with 0.1M hydrochloric acid and measured absorbance at 700 nm with tannic acid (TA) as a standard. The tannin content was determined to be TA equivalent (mg TA/g).

Antioxidant activity

Ferric-Reducing Ability of Plasma (FRAP) Assay

A 3.6 mL FRAP solution was incubated at 37°C with 0.4 mL of distilled water for five minutes. Subsequently, the FRAP solution was combined with 80 µL of the formulation and incubated for 10 minutes at 37°C. The absorbance of the reaction mixture was measured at 593 nm. FeSO₄·7H₂O at concentrations of 0.1, 0.4, 0.8, 1.0, 1.12, and 1.5 mM was used for the calibration curve, and the absorbance values were determined in the same manner as those of the sample solutions [16,17].

Nitric Oxide (NO) Assay

Garrat (1964) reported that the Griess-Ilosvay reaction could be used to determine the inhibition of NO radicals. N-(1-naphthyl)ethylenediamine dihydrochloride (0.1% w/v) was used instead of 1-naphthylamine (5%). The reaction mixture (3 mL), containing rutin (0.5 mL), sodium nitroprusside (10 mM, 2 mL), and phosphate-buffered saline (0.5 mL), was incubated with an extract (10 µg to 50 µg) for 150 minutes. The above reaction mixture (0.5 mL) and sulfanilic acid reagent (1 mL of 0.33% in 20% glacial acetic acid) were combined, and the mixture was left to stand for five minutes to complete diazotization. N-(1-naphthyl)ethylenediamine dihydrochloride (1 mL) was then added, stirred, and allowed to stand for 30 minutes at 25°C. Under diffuse light, a pink chromophore was formed. The absorbance of these solutions was measured at 540 nm against matching blank solutions, with rutin employed as a standard [18].

2,2-Azino-Bis-3-Ethylbenzothiazoline-6-Sulphonic Acid (ABTS) Assay

We measured antioxidant activity using the ABTS assay, as previously reported, with slight modifications to the protocol. A solution of 1.05 mM ABTS (in 50% ethanol) and 2.45 mM potassium persulfate (in distilled water) was used to generate the ABTS radical cation (ABTS•+). The mixture was kept in a cold condition for 24 hours. Afterwards, 50% ethanol was added to the reagent until the absorbance reached 1.0 ± 0.02 at 734 nm. In 96-well microplates, 20 µL of formulations at each concentration were added to 250 µL of ABTS solution. Ethanol (20 µL) was used as a blank. The absorbance was measured at 734 nm using a microplate reader (SpectraMax 190 Microplate Reader, Molecular Devices, Sunnyvale, USA). The results were expressed as IC50 (the concentration capable of reducing 50% of free radicals), compared to the antioxidant activity of quercetin, butylated hydroxy-anisole (BHA) and butylated hydroxy-toluene (BHT) [19].

Anti-inflammatory activity

Human Red Blood Cell (HRBC) Membrane Stabilization Assay

RBC suspension was prepared by placing a sample of freshly drawn human blood into a sterile tube containing an anticoagulant. The RBCs were separated from the other components by centrifugation at 1000 rpm for 10 minutes at room temperature. The RBCs were washed three times with phosphate-buffered saline (PBS), and the supernatant was discarded. The RBCs were then reconstituted in Tris-HCl buffer to yield a 10% (v/v) RBC suspension. One millilitre of the RBC suspension was added to each centrifuge tube using a micropipette. Various concentrations of *Ixora* and *Rhododendron* extracts were added to the tubes, and the contents were thoroughly mixed. The tubes were incubated at 37°C for 30 minutes under controlled conditions. The absorbance of the supernatant was measured at 540 nm using UV-Vis spectrophotometry. The following formula was used to determine membrane stabilization [20]:

\[ \text{Membrane Stabilization (%)} = \left( \frac{\text{OD control} - \text{OD sample}}{\text{OD control}} \right) \times 100 \]

Here, "OD sample" represents the absorbance of the RBC suspension in the presence of the test compound, while "OD control" refers to the absorbance of the RBC suspension in the absence of the test compound(s).

Heat-Induced Hemolysis

A portion of 5 mL of isotonic buffer containing 10-50 μg/mL of ethanolic crude extract was placed into each of two sets of centrifuge tubes. Another tube, containing the same volume of the vehicle, was used as a control. After adding 50 μL of RBC suspension to each tube, the tubes were inverted and gently mixed. One set of tubes was incubated in a water bath at 54°C for 20 minutes, while the other set was stored in an ice bath at a temperature range of 0 to 5°C. The mixture was then centrifuged for five minutes at 5000 rpm, and the absorbance was measured at 560 nm using a spectrophotometer. Diclofenac sodium (DS) was used as a reference standard at 10-50 μg/mL [21].

Cytotoxic effect

Brine Shrimp Lethality Assay

Two grams of iodine-free salt were dissolved in 200 mL of distilled water. Each well of a six-well enzyme-linked immunosorbent assay (ELISA) plate was filled with 10-12 mL of the saline solution. Subsequently, 10 nauplii were carefully added to each well, which was labelled with varying concentrations (20, 40, 60, 80, 100 µg/mL). Samples were then added according to their respective concentration levels, and the plates were incubated for 24 hours [22,23]. After incubation, the ELISA plates were examined to determine the number of live nauplii, which was calculated using the following formula:

\[ \text{Viability (%)} = \left( \frac{\text{Number of live nauplii}}{\text{Total number of nauplii}} \right) \times 100 \]

Zebrafish Embryonic Toxicity Test

Zebrafish embryos were incubated at 26°C for four hours post-fertilization, and healthy embryos were randomly selected and placed in six-well plate cultures with 0.2 mL of culture water at the spherical stage. Formulations at concentrations of 1 mM (5, 10, 20, 40, and 80 µg/mL) were introduced to each well. The experiments were conducted in triplicate, with embryos in the culture medium serving as the control group. The plates were then incubated at 26°C to observe the development of zebrafish larvae and embryos at various stages of fertilization. The percentages of hatching and death were determined every 12 hours based on the total number of surviving embryos. Any embryonic malfunctions related to the formulation were identified under a microscope [24,25].

Docking study

For the preparation of ligand structures, the two-dimensional (2D) structures of ursolic acid, quercetin, and anthocyanin were obtained from PubChem. The ligands were then optimized using molecular mechanics methods to achieve low-energy conformations. The crystal structures of proteins such as nuclear factor kappa B (NF-κB), cyclooxygenase-1 (COX-1), and cyclooxygenase-2 (COX-2) were retrieved from the Protein Data Bank (PDB). Co-crystallized ligands and water molecules were removed from the structures, and missing hydrogen atoms and charges were added to the protein structures. Grids were generated around the active sites of NF-κB, COX-1, and COX-2 using the coordinates of their respective binding sites, encompassing the key residues involved in ligand binding. Molecular docking was performed using software such as AutoDock Vina (Scripps Research Institute, La Jolla, USA). Docked poses were ranked based on scoring functions including binding energy, docking score, and intermolecular interactions. The top-ranked poses were visually inspected to analyze the orientation and binding interactions of the ligands within the active sites of NF-κB, COX-1, and COX-2. Fundamental interactions, such as hydrogen bonds, hydrophobic contacts, and π-π stacking, were identified and compared across the three proteins [26].

## Results

Quantitative phytochemical analysis

Quantitative analysis was conducted for phytochemicals such as alkaloids, phenols, flavonoids, saponins, and tannins, with the results presented in Table 1. It was determined that the ethanol extracts of *I. coccinea *and *R. arboreum* flowers generally had higher phytochemical content compared to their aqueous extracts.

**Table 1:** Quantitative analysis of phytochemicals in Ixora coccinea and Rhododendron arboreum Data are expressed as standard equivalent per gram of dry extract; represented in the average of replicates ± standard deviation (AVG ± SD). AT: atropine; GA: gallic acid; CN: catechin; DI: diosgenin; TA: tannic acid; RhW: Rhododendron water extract; RhE: Rhododendron ethanolic extract; IxW: Ixora water extract; IxE: Ixora ethanolic extract

Phytochemical constituents	Alkaloid (mg AT/g)	Phenol (mg GA/g)	Flavonoid (mg CN/g)	Saponin (mg DI/g)	Tannin (mg TA/g)
RhW	0.80 ± 0.54	35.81 ± 0.45	51.23 ± 0.12	1.29 ± 0.87	49.84 ± 0.65
RhE	12.39 ± 0.75	56.12 ± 0.91	64.56 ± 0.2	1.98 ± 0.74	37.42 ± 0.77
IxW	11.76 ± 0.45	61.27 ± 0.5	58.31 ± 0.78	21.02 ± 0.54	11.06 ± 0.56
IxE	17.83 ± 0.12	59.78 ± 0.23	64.23 ± 0.91	0.99 ± 0.64	13.98 ± 0.93

The alkaloid content in the ethanol extracts was found to be higher than in the aqueous extracts (RhE - 12.39 mg AT/g and IxE - 17.83 mg AT/g vs. RhW - 0.80 mg AT/g and IxW - 11.76 mg AT/g). The phenol content was higher in the aqueous extract of *I. coccinea* compared to the ethanolic extract (61.27 mg GA/g vs. 56.78 mg GA/g). Conversely, the ethanolic extract of *R. arboreum *contained a higher concentration of phenolics compared to the water extract (56.12 mg GA/g vs. 35.81 mg GA/g). The ethanol extracts of both flowers had significantly higher flavonoid content compared to the aqueous extracts (RhE - 64.56 mg CN/g and IxE - 64.23 mg CN/g vs. RhW - 51.23 mg CN/g and IxW - 58.31 mg CN/g). Based on various quantitative phytochemical analyses, both solvent types exhibited high levels of flavonoid content in the flowers.

The *R. arboreum* water extract had higher tannin content (RhW - 49.84 mg TA/g) compared to its ethanolic extract (RhE - 37.42 mg TA/g) and the *I. coccinea* extracts (IxW - 11.06 mg TA/g and IxE - 13.98 mg TA/g). The ethanol extract of *I. coccinea* (IxE - 0.99 mg DI/g) contained a marginal amount of saponins. In contrast, the water extract of *I. coccinea *had a higher saponin content (21.02 mg DI/g). For *R. arboreum*, the saponin levels were relatively low, with 1.98 mg DI/g in the ethanol extract and 1.29 mg DI/g in the water extract.

Assessment of free radical scavenging activity of the formulation

FRAP Assay

**Figure 2:**
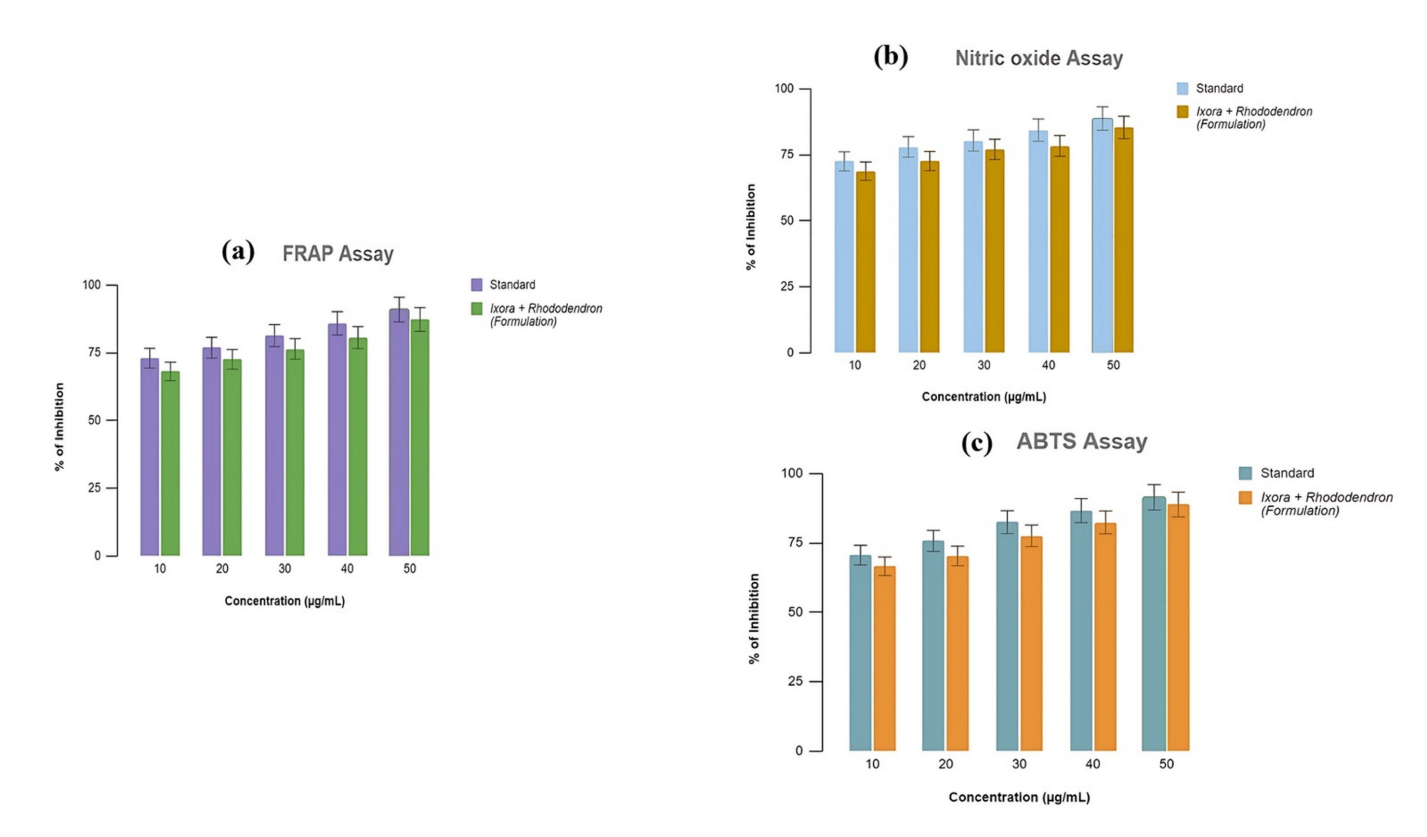
The formulation exhibits potent inhibition of reactive oxygen species (ROS), showing a significant dose-dependent response in the FRAP assay, with 87.24% inhibition at the highest concentration of 50 µg/mL (a). In the NO assay (b), the formulation demonstrated peak inhibition of 85.27% at 50 µg/mL. Additionally, the ABTS assay (c) revealed substantial inhibition of 66.54% at a lower concentration of 10 µg/mL, underscoring the formulation's effective antioxidant activity across various concentrations. FRAP assay: ferric-reducing ability of plasma assay; NO assay: nitric oxide assay; ABTS assay: 2,2-azino-bis-3-ethylbenzothiazoline-6-sulphonic acid assay

The prepared medium, containing varying concentrations of *Ixora* and *Rhododendron* ranging from 10 to 50 µg/mL, demonstrated notable ROS inhibition. The percentage inhibition at these concentrations was 66.54%, 70.28%, 77.49%, 82.33%, and 88.76%, respectively, with an IC50 value of 2.81 µg/mL. This indicates that the formulation exhibited significant ROS-scavenging activity (Figure 2a) compared to the standard FeSO₄, which showed an IC50 value of 4.04 µg/mL.

NO Assay

The formulation of *Ixora* and *Rhododendron* at concentrations of 10, 20, 30, 40, and 50 µg/mL showed a progressive increase in ROS inhibition with percentage inhibitions of 68.7%, 72.6%, 76.9%, 78.3%, and 85.3%, respectively. The IC50 value of the formulation was determined to be 3.79 µg/mL, demonstrating comparable activity to the standard rutin, which exhibited an IC50 value of 4.92 µg/mL (Figure 2b).

ABTS Assay

The formulated solution of *Ixora* and *Rhododendron* at varying concentrations of 10, 20, 30, 40, and 50 µg/mL showed ROS inhibition of 68.74%, 72.56%, 76.99%, 78.31%, and 85.27%, respectively, with an IC50 value of 1.79 µg/mL. These results were compared to the predetermined combination of quercetin, BHA, and BHT, which had an IC50 value of 2.95 µg/mL. The research demonstrated that the combination of *Ixora* and *Rhododendron* produces similar activity (Figure 2c).

Assessment of membrane stabilization potential of the formulation

**Figure 3:**
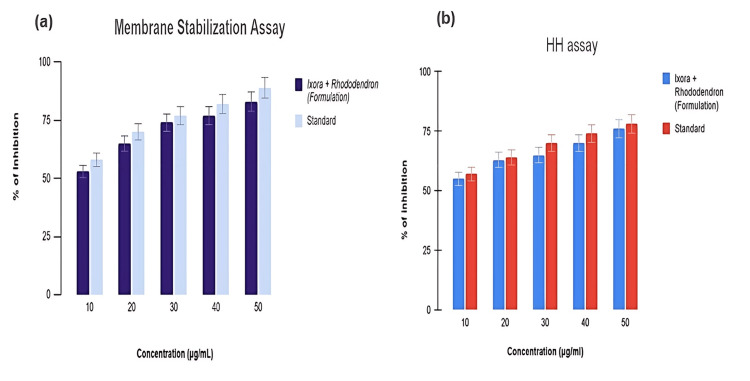
Membrane stabilization by the formulation shows a significant increase in efficacy in a concentration-dependent manner (a). In the heat-induced hemolysis model (b), the formulation achieves significant inhibition, reaching a peak of 76% at the highest concentration of 50 µg/mL, closely comparable to the standard's inhibition rate of 78%. HH assay: heat-induced hemolysis assay

The degree of stabilization in the membrane stabilization assay increased steadily with various concentrations. At the first dilution (10 µg/mL), the formulation and the standard demonstrated comparable percentage inhibition, with the formulation showing approximately 53% and the standard (DS) showing 58%. However, at higher concentrations, both the formulation and the standard exhibited more pronounced effects, with approximately 83% inhibition for the formulation and 89% inhibition for the standard. The IC50 values were 4.05 µg/mL for the formulation and 4.16 µg/mL for the standard (Figure 3a). The anti-inflammatory effect was also assessed using the heat-induced hemolysis method, as shown in Figure 3b. It was observed that the formulation's inhibitory effect increased with higher concentrations, yielding an IC50 value of 5.6 µg/mL. This effect was comparable to that of the standard, which had an IC50 value of 3.61 µg/mL at the same concentration.

Brine Shrimp Lethality Assay

**Figure 4:**
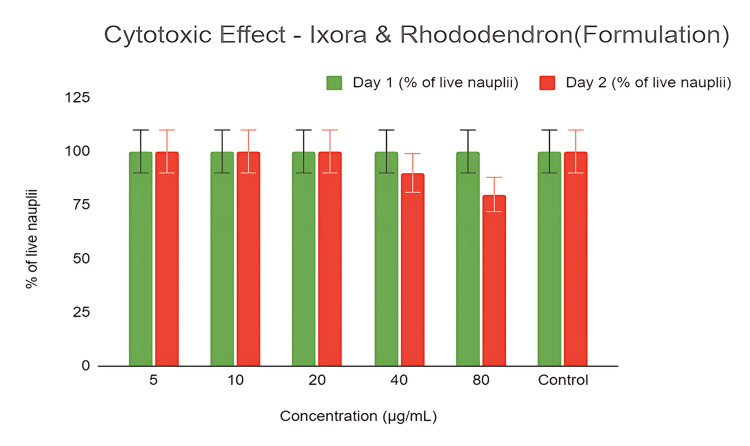
The brine shrimp lethality assay revealed minimal cytotoxicity of the formulation within the tested range of 5 to 80 µg/mL. Concentrations up to 20 µg/mL showed no mortality, while higher concentrations of 40 and 80 µg/mL resulted in 10% and 20% mortality rates, respectively.

The brine shrimp lethality assay was employed to test and analyze cytotoxicity levels, providing an effective method to assess the cytotoxic effects of the prepared formulation. The graph illustrates the cytotoxicity levels of the formulation. Ten nauplii were added to formulations with concentrations of 5, 10, 20, 40, and 80 µg/mL in each of six wells, along with a control group. No nauplii mortality was observed by day 2 at concentrations of 5, 10, and 20 µg/mL. Cytotoxicity became evident at 40 µg/mL, with a 10% mortality rate, while 80 µg/mL showed a 20% mortality rate. The control group's nauplii remained viable on both days (Figure 4). These results suggest minimal cytotoxicity, with an LC50 of 30.5 µg/mL within the tested concentration range (5 to 80 µg/mL).

Zebrafish Embryonic Toxicology Test

**Figure 5:**
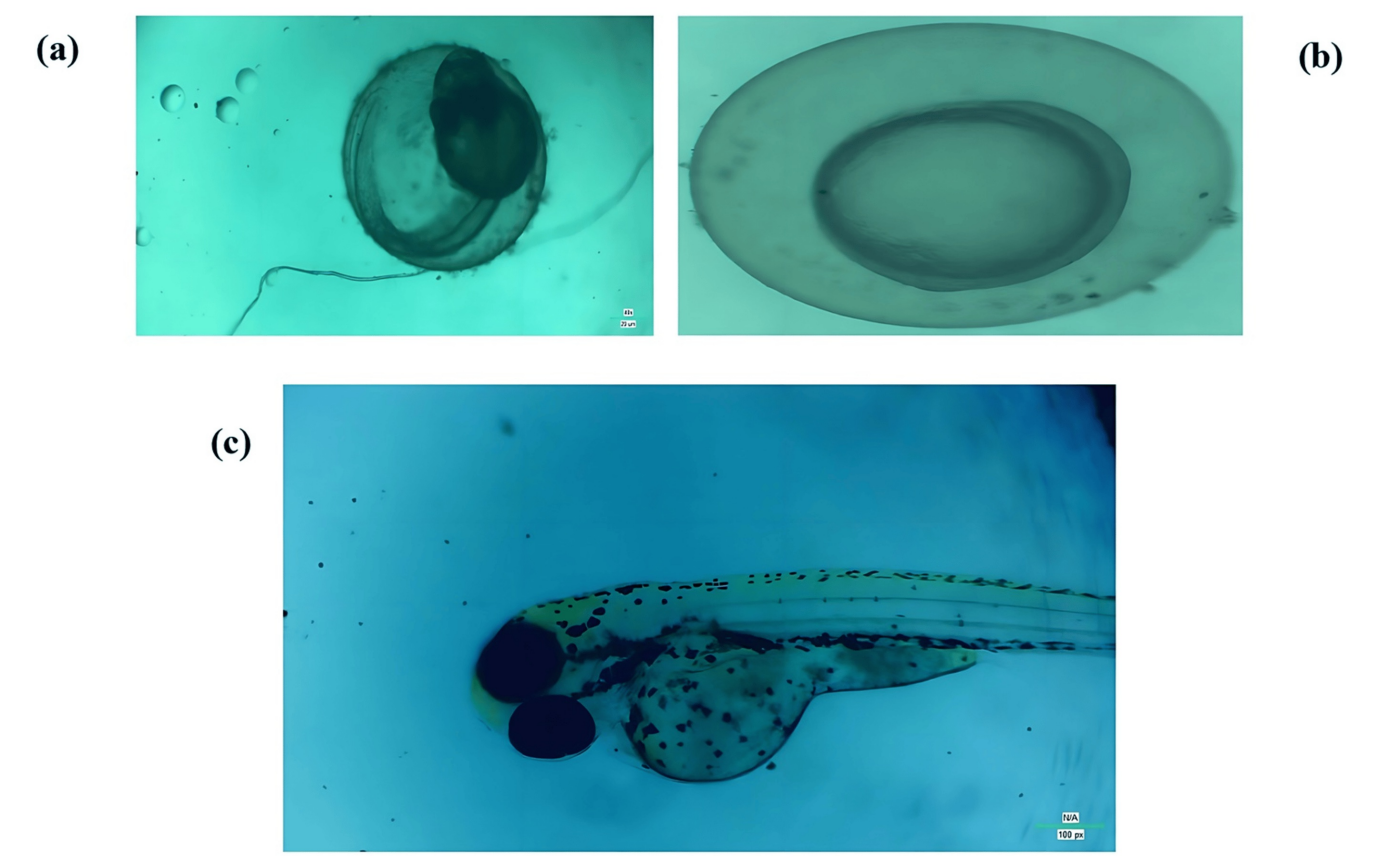
Images displaying zebrafish embryos exposed to the formulation at different developmental stages: (a) 24 hours post-fertilization, showing early developmental features; (b) 48 hours post-fertilization, indicating further organ development and tail elongation; and (c) 72 hours post-fertilization, with visible pigmentation and a more defined structure.

The zebrafish embryonic toxicity test is a more sensitive method compared to the brine shrimp lethality assay. In this study, the toxicity levels of the *Ixora* and *Rhododendron* formulation at various concentrations (5, 10, 20, 40, and 80 µg/mL) were observed under a microscope at different developmental stages: 24 hours post-fertilization (a), 48 hours post-fertilization (b), and 72 hours post-fertilization (c) (Figures 5a-5c).

**Figure 6:**
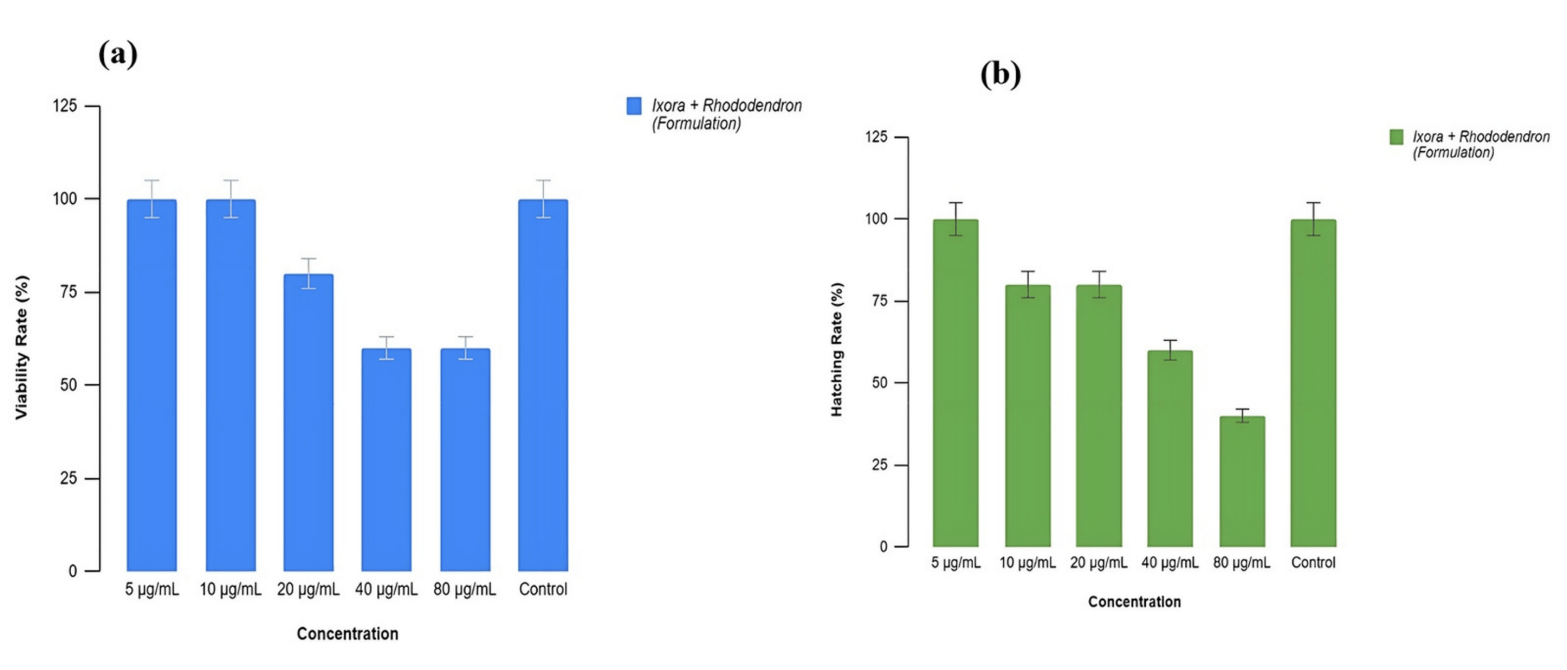
(a) depicts the dose-dependent mortality rates of zebrafish embryos and larvae, with an LC50 concentration of 82.4 µg/mL, indicating increased mortality at higher doses; (b) illustrates the hatching rates, with an LC50 of 53.33 µg/mL, suggesting a decline in hatching success as the formulation concentration increases, reflecting a dose-dependent increase in toxicity. LC50: lethal concentration 50%

We monitored the mortality rates of zebrafish embryos and larvae at regular intervals, observing zero mortality on day 1. However, by day 2, treated embryos showed a dose-dependent increase in mortality under laboratory conditions. Specifically, at a concentration of 20 µg/mL, viability dropped to below 80%, while concentrations of 40 and 80 µg/mL resulted in a more significant reduction in viability, although the viability of nauplii remained above 50%. The safety and potential of the formulations were illustrated by the graph (Figure 6a), which showed that viability decreased below 50% at concentrations beyond 80 µg/mL, indicating an LC50 concentration of 82.4 µg/mL.

Additionally, we examined the hatching rates of embryos exposed to varying concentrations of the formulations ranging from 5 to 80 µg/mL. On day 1, hatching rates were 0% at all tested concentrations. By day 2, the hatching rate was reduced to less than 50% at 80 µg/mL, with an LC50 value of 53.33 µg/mL. These findings suggest a proportional increase in toxicity with higher concentrations, as shown in Figure 6b. The formulation maintained normal embryo morphology without malformations throughout the test duration (Figures 5a-5c).

Molecular docking

Binding Affinities and Interactions With NF-κB

**Figure 7:**
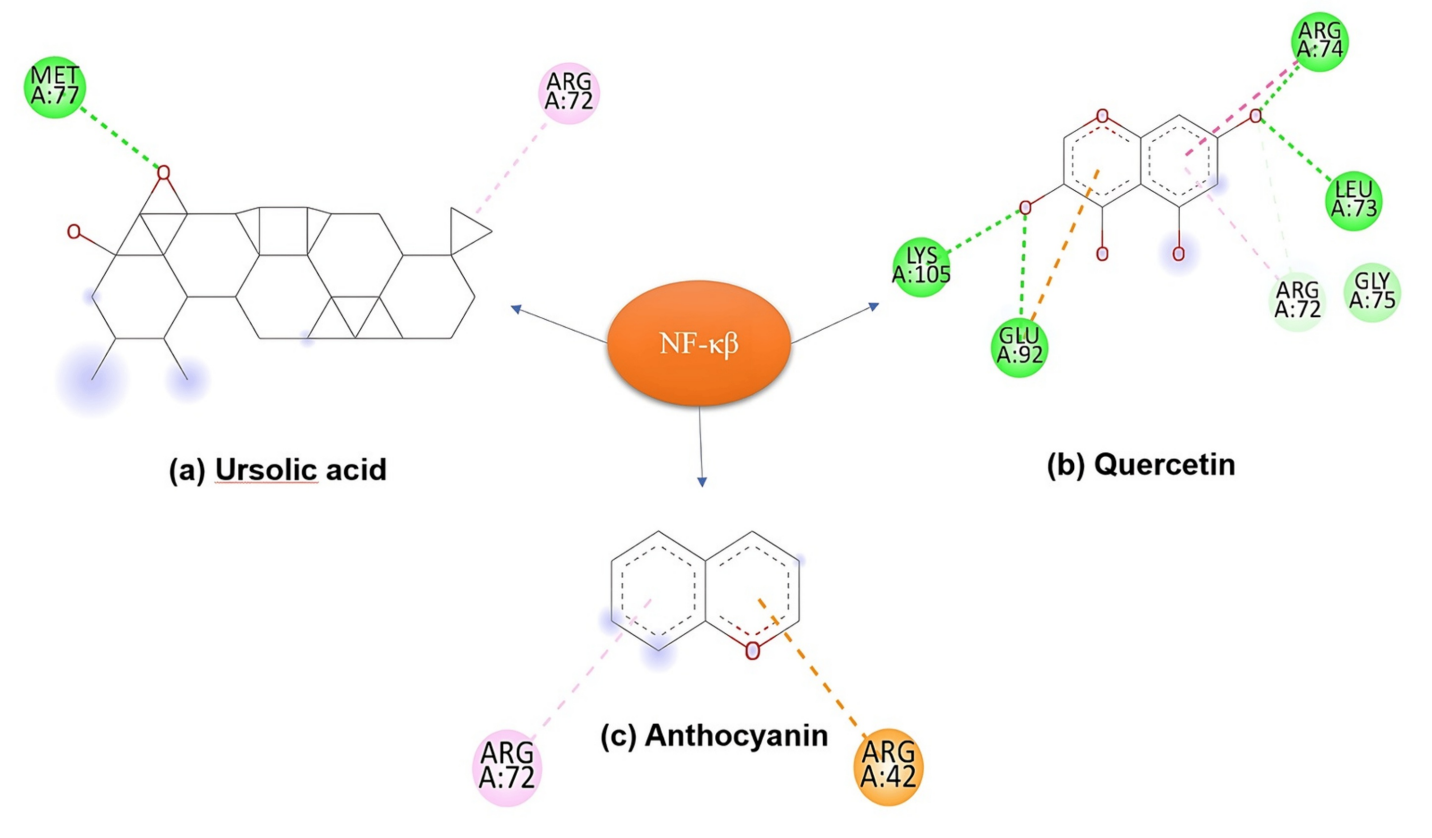
On docking with NF-κB: (a) Ursolic acid displayed moderate binding affinity, primarily engaging in hydrophobic interactions. (b) Quercetin exhibited strong binding affinity, forming hydrogen bonds with key residues in the active site. (c) Anthocyanin showed the weakest binding affinity, with fewer favourable interactions compared to ursolic acid and quercetin. NF-κB: nuclear factor kappa B

Molecular docking analysis revealed that quercetin exhibited a robust binding affinity towards NF-κB, forming hydrogen bonds with key residues in the active site, thus highlighting its potential as a promising inhibitor of NF-κB-mediated signalling pathways. In contrast, ursolic acid demonstrated moderate binding affinity, primarily through hydrophilic and hydrophobic interactions. Anthocyanin, while still interacting with the NF-κB active site, showed the weakest binding affinity among the three ligands, indicating its lesser potential as an NF-κB inhibitor (Figure 7).

Binding Affinities and Interactions With COX-1

**Figure 8:**
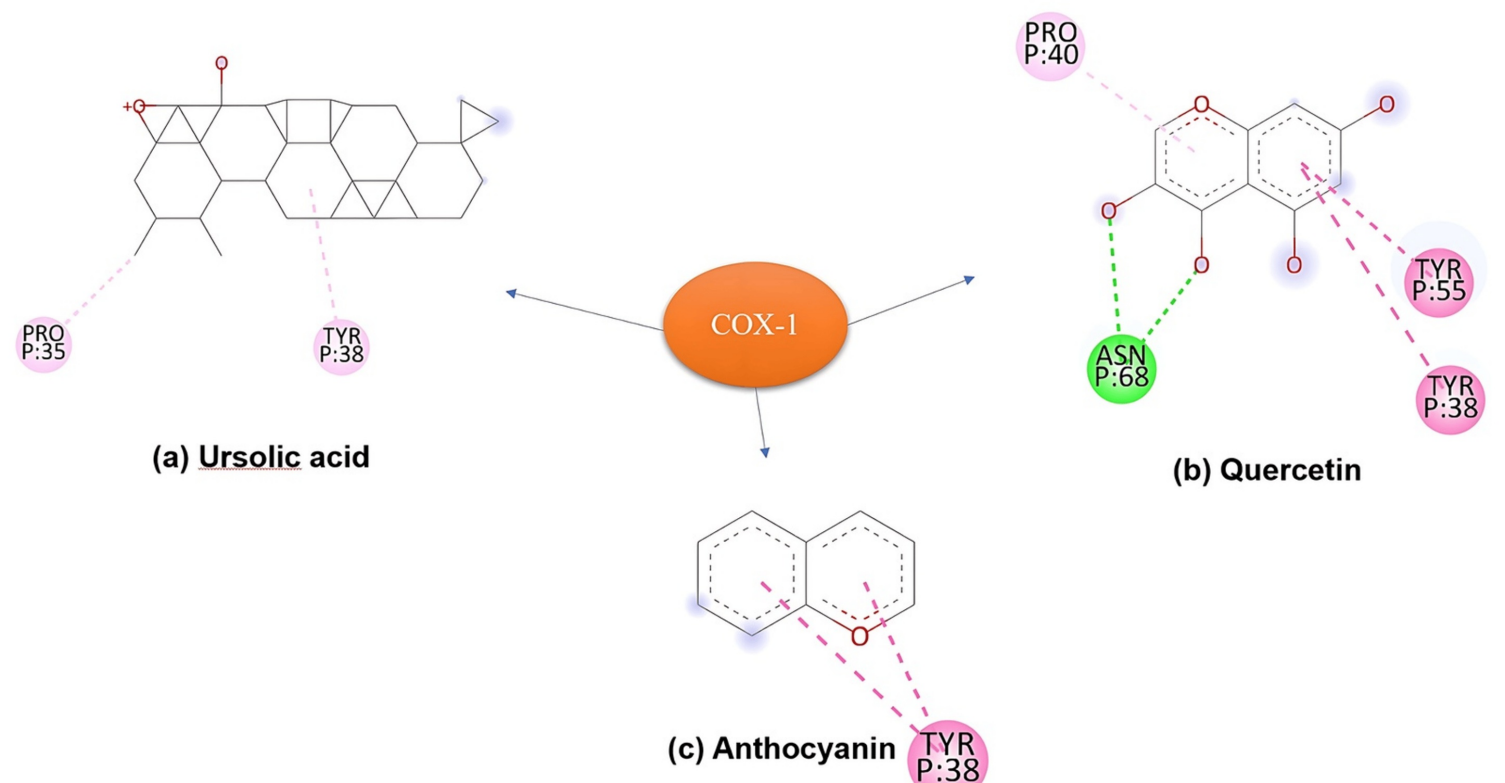
On docking with COX-1: (b) Quercetin demonstrated the highest binding affinity among the tested ligands, forming hydrogen bonds with key residues in the COX-1 active site. (a) Ursolic acid and (c) anthocyanin showed weaker binding affinities, predominantly interacting through hydrophobic contacts. COX-1: cyclooxygenase-1

Quercetin demonstrated the highest binding affinity towards COX-1, forming hydrogen bonds with key active site residues (Figure 8), suggesting its potential as an effective COX-1 inhibitor with possible anti-inflammatory properties. In comparison, ursolic acid and anthocyanin exhibited weaker binding affinities, driven mostly by hydrophobic interactions, notably with residue TYR P:38. Despite their lower binding strengths, these interactions may still play a role in their pharmacological effects on COX-1, offering avenues for further exploration.

Binding Affinities and Interactions With COX-2

**Figure 9:**
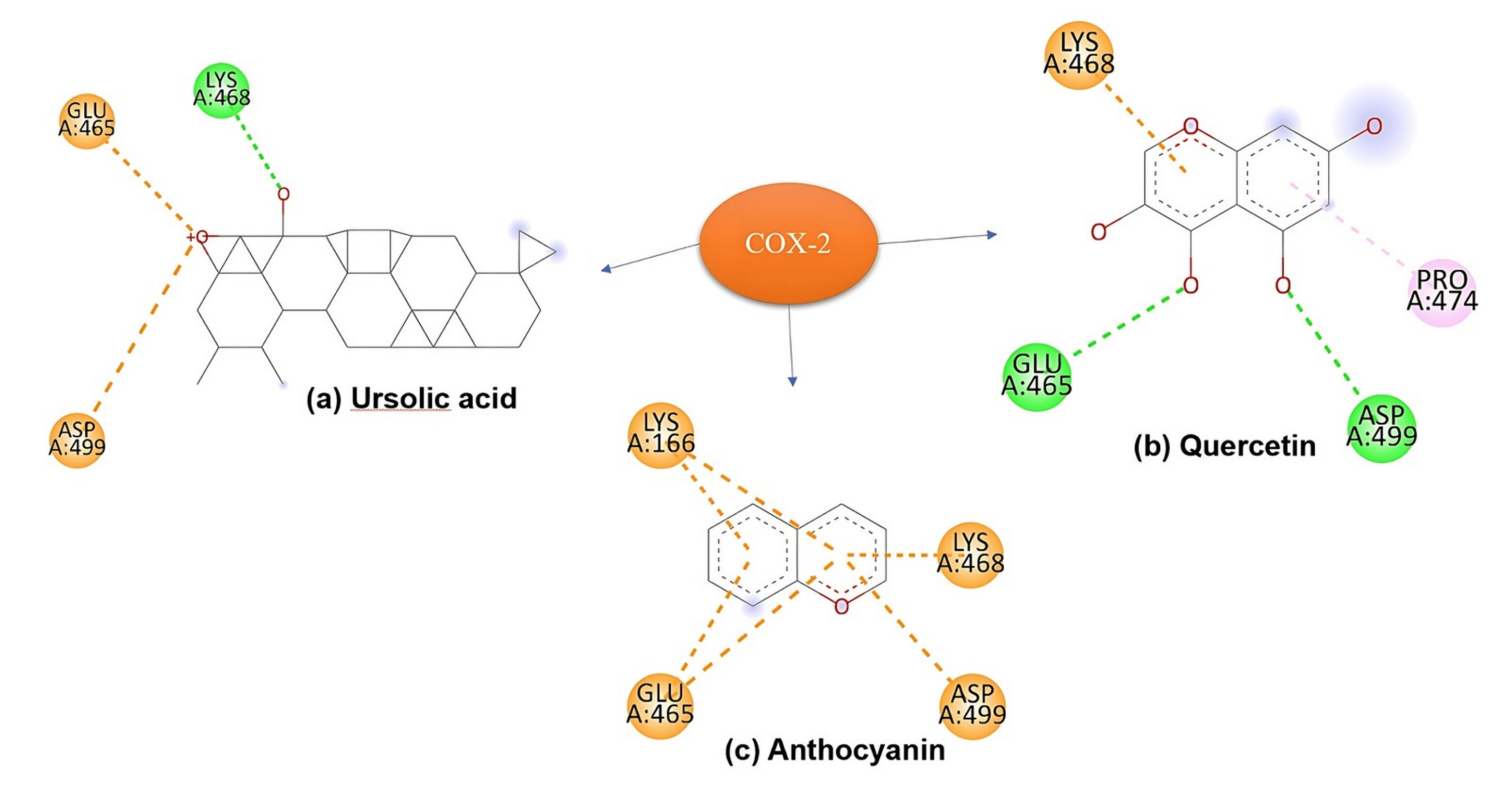
On docking with COX-2: (b) Quercetin displayed the strongest binding affinity, forming multiple hydrogen bonds and hydrophobic interactions with key residues in the COX-2 active site. (a) Ursolic acid showed moderate binding affinity, primarily interacting through hydrophobic contacts, while (c) anthocyanin exhibited weaker binding affinity than both quercetin and ursolic acid, with fewer favourable interactions observed. COX-2: cyclooxygenase-2

Quercetin exhibited the strongest binding affinity towards COX-2, forming multiple hydrogen bonds and hydrophobic interactions with critical active site residues (Figure 9), positioning it as a promising COX-2 inhibitor with potential therapeutic applications in treating inflammation. Ursolic acid showed a moderate binding affinity, primarily driven by hydrophobic interactions. In contrast, anthocyanin demonstrated the weakest binding affinity, with fewer favourable interactions within the COX-2 active site, indicating its comparatively lower potential as a COX-2 inhibitor.

## Discussion

During the pathological conditions of oxidative stress, there can be a discernible rise in the production of RNS and ROS, coupled with lower levels of antioxidant status, which results in changes in membrane lipids, proteins, and nucleic acids. The degradation of these biomolecules by ROS/reactive nitrogen species (RNS) is associated with ageing and other clinical processes, including neurodegenerative diseases, ischemia-reperfusion injury, cancer, and atherosclerosis [27].

The antioxidant potency is measured by the FRAP assay, which examines the percentage reduction of ferric ion to ferrous ion in the presence of a reducing agent. The antioxidant potency of the formulation was evidenced through the colour shift to violet due to electron transfer. The formulation exhibited 68.09% inhibition at a dose of 10 µg/mL and 86.9% inhibition at 50 µg/mL, with an IC50 value of 2.81 mg/mL. Compared to ferrous sulphate, a typical oxidizing agent, the formulation exhibited 86.9% inhibition at 50 µg/mL. The ethanol extracts of *I. coccinea* and *R. arboreum* showed significant ferric-reducing antioxidant strength, as evidenced by the concentration-dependent rise in absorbance values, similar to the findings of a study that proposed the antioxidant potential of *Bauhinia rufescence* Lam leaf extract [28]. Further research on the NO antioxidant assay assessed the degree to which antioxidants neutralize RNS, such as NO2 [29]. In this assay, the sample is exposed to a solution containing NO2, and the ability of antioxidants to counteract its effects is assessed. After incubation, the remaining NO2 concentration is measured, indicating the antioxidant capacity of the sample. The results indicate that the formulation, at the highest concentration, can combat oxidative stress with 85.27% inhibition, comparable to rutin solution, which showed 85.27% inhibition with an IC50 value of 3.79 mg/mL.

Antioxidant efficiency in decreasing chromophoric reactive radical cation ABTS•+ ROS has also been assessed in vitro using the ABTS radical scavenging method [30]. By donating electrons, antioxidants in a sample neutralize ABTS•+, lowering absorbance. This decline correlates with the sample's antioxidant activity. Results are expressed as quercetin equivalents for comparison. The prepared formulation provided valuable insights into the antioxidant capacity of compounds at 50 µg/mL, showing 85.27% inhibition, comparable to the standard drug (88.76%) at the same concentration, with an IC50 value of 1.79 mg/mL.

An imbalance between the body’s capacity to eliminate ROS with antioxidants and the generation of ROS during embryonic development leaves the embryo particularly vulnerable to oxidative stress. Damage to DNA, oxidation of proteins, peroxidation of lipids, and disturbance of cellular functions are all consequences of oxidative stress that can adversely influence embryonic development. Membrane stabilization prevents the leakage of fluids and serum proteins into tissues, mediated by inflammatory factors that increase membrane permeability [31]. The combined formulation may stabilize RBC membranes by preventing the release of lytic enzymes and other inflammatory mediators. At the highest dose (50 μg/mL), the combination of *Ixora* and *Rhododendron* flowers demonstrated nearly equivalent activity (IC50 value of 4.05 mg/mL) to that of the reference standard.

Quercetin exhibited robust binding affinities towards NF-κB, COX-1, and COX-2, forming hydrogen bonds and hydrophobic interactions with critical residues within the active sites. This suggests quercetin's potential as a promising inhibitor of NF-κB-mediated signalling pathways and COX enzymes, highlighting its anti-inflammatory and anticancer properties. Supporting evidence suggests a direct correlation between quercetin's binding affinity to various biomolecules implicated in cancer progression, such as NF-κB, COX enzymes, YY1, and DNA, and its potent cytotoxicity against a broad range of cancer cell lines. Quercetin's binding interactions appear to be a key mechanism underlying its anticancer effects; thus, further study is warranted to explore the anticancer potential of the formulations.

To develop interventions and preventive measures to lower the risk of developmental abnormalities, it is crucial to understand how antioxidants protect embryonic development. In this study, brine shrimp lethality was employed to measure and appraise the degree of cytotoxicity. In each of the six wells, 10 nauplii were added to the formulations, with concentrations of 5, 10, 20, 40, and 80 mg/mL, along with a control group. On day 2, no nauplii mortality was observed at concentrations of 10 and 20 mg/mL. Cytotoxicity was observed at an exposure level of 40 mg/mL, with a 10% mortality rate. On days 1 and 2, all of the nauplii in the control group were alive. The obtained data indicate that the concentrations (<80 µg/mL) employed for analysis showed minimal cytotoxicity. The viability of nauplii at concentrations of 20, 40, and 80 mg/mL was more than 50%. However, the viability decreased to less than 50% at concentrations greater than 80 mg/mL. Based on the results, the LC50 concentration was found to be approximately 30.5 mg/mL. Thus, the formulations have been shown to have relatively low toxicity levels, even at higher concentrations.

The zebrafish embryonic toxicity assay further supports the cytotoxicity assessment conducted via the brine shrimp lethality assay. Low toxicity levels were found at the concentrations examined in the current investigation. On day 1, no mortality was observed. Under laboratory conditions, on day 2, the treated embryos showed a dose-dependent increase in mortality. The viability of the embryos was 60% at a concentration of 80 mg/mL, with an LC50 value of 82.4 µg/mL. The hatching rates of embryos exposed to various formulation concentrations, ranging from 10 to 80 mg/mL, are shown in the graph (Figure 6b). The hatching rate was determined to be zero on the first day. On day 2, the egg hatching rate was reduced to 80% at a concentration of 10 mg/mL. There was a noticeable drop in the hatching rate to around 60% at 50 mg/mL. It was found that the formulation had an LC50 of 53.33 µg/mL and a hatching rate slightly above 50% at the higher concentration of 80 mg/mL. Therefore, based on the results, toxicity increases proportionally with formulation concentration.

The findings of this study demonstrate the formulation's superior inhibitory capacity against standard inflammatory mediators, even under conditions of heat-induced hemolysis, by effectively stabilizing the cell membrane. Furthermore, the research confirms the synergistic benefits of combining *I. coccinea* and *R. arboreum* in traditional medicine for conditions associated with oxidative stress. Throughout the study, no abnormalities were observed in the embryos during the investigation, and they retained their normal morphology. This advances our understanding of factors influencing fetal health and embryo development.

Two basic limitations of this study are the limited range of concentrations tested and the use of only in vitro and embryonic models. Future studies will address these by including a broader range of concentrations to establish a more comprehensive dose-response relationship and incorporating mammalian models to better assess physiological relevance and potential side effects in more complex biological systems.

## Conclusions

This study demonstrates the potent antioxidant and anti-inflammatory effects of* I. coccinea* and* R. arboreum* in a combined formulation, even at higher concentrations. The formulations effectively inhibited ROS and stabilized cell membranes, exhibiting significant antioxidant capacity comparable to standard treatments. Cytotoxicity assays using brine shrimp and zebrafish embryos confirmed low toxicity, with high viability and normal embryonic development observed at tested doses. The synergistic benefits of these combined extracts support their potential use in managing oxidative stress-related conditions, providing a basis for further research and potential clinical applications.
